# An infant with coronavirus disease 2019 in China: A case report

**DOI:** 10.1097/MD.0000000000021359

**Published:** 2020-07-17

**Authors:** Wen Cao, Gang Mai, Zhen Liu, Haoyuan Ren

**Affiliations:** People's Hospital of Deyang City, Sichuan, China.

**Keywords:** coronavirus disease 2019, infant, oropharyngeal swab, reverse transcription-polymerase chain reaction, severe acute respiratory syndrome coronavirus 2

## Abstract

**Rationale:**

In December 2019, an outbreak of coronavirus disease 2019 (COVID-19) occurred in Wuhan, China. The initial epidemiological investigations showed that COVID-19 occurred more likely in adults, with patients younger than 10 years old accounting for less than 1% of the total number of confirmed cases, and infant infections were more rare. In our case, we present an infant who was only 35 days old when he was tested positive for COVID-19.

**Patient concerns:**

In this report, a 35 day-old male infant with atypical symptoms had close contact with 2 confirmed patients of COVID-19 who were his grandmother and mother.

**Diagnosis:**

The patient was diagnosed as COVID-19 after his oropharyngeal swab tested positive for severe acute respiratory syndrome coronavirus 2 by reverse transcription-polymerase chain reaction assay.

**Interventions:**

The therapeutic schedule included aerosol inhalation of recombinant human interferon α-2b and supportive therapy.

**Outcomes:**

Two consecutive (1 day apart) oropharyngeal swabs tested negative for severe acute respiratory syndrome coronavirus 2; then, the patient was discharged on February 27, 2020.

**Lessons:**

Strengthening infants’ virus screening in families with infected kins is important for early diagnosis, isolation, and treatment when symptoms are atypical. The infectivity of infants with mild or asymptomatic COVID-19 should not be ignored because this may be a source of transmission in the community.

## 1 Introduction

In December 2019, an outbreak of Coronavirus disease 2019 (COVID-19) occurred in Wuhan, China. As of April 17, 2020, a total of 82,719 COVID-19 cases in China had been confirmed.^[1]^ The rapid spread of the epidemic indicated that the Severe Acute Respiratory Syndrome Coronavirus 2 (SARS-CoV-2) had a strong transmissibility in human populations. The initial epidemiological investigations showed that COVID-19 occurred more likely in adults,^[2,3]^ with patients younger than 10 years old accounting for less than 1% of the total number of confirmed cases,^[4,5]^ and infant infections were more rare. According to previous reports and the present case, infants can be infected by SARS-CoV-2. A study analyzed the clinical characteristics of 9 infants infected by SARS-CoV-2 between December 8, 2019, and February 6, 2020. The youngest was 1 month and 26 days old, and the oldest was 11 months old. All infants had mild or no clinical symptoms, and none of them required intensive care or mechanical ventilation or had any severe complications,^[6]^ which was similar to the previously reported in severe acute respiratory syndrome (SARS) and Middle East respiratory syndrome.^[7,8]^

## 2 Case presentation

**Figure 1:**
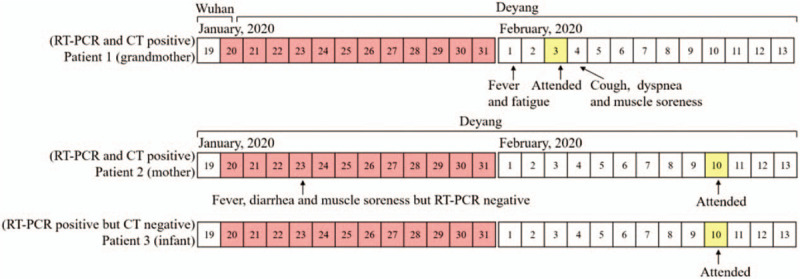
Chronology of symptom onset of the 3 patients and their contacts in Wuhan Dates filled in red are the dates when the 3 patients 1 to 3 had close contacts with each other. Dates filled in yellow are the dates when the SARS-CoV-2 RT-PCR showed positive. RT-PCR = reverse transcription-polymerase chain reaction.

**Figure 2:**
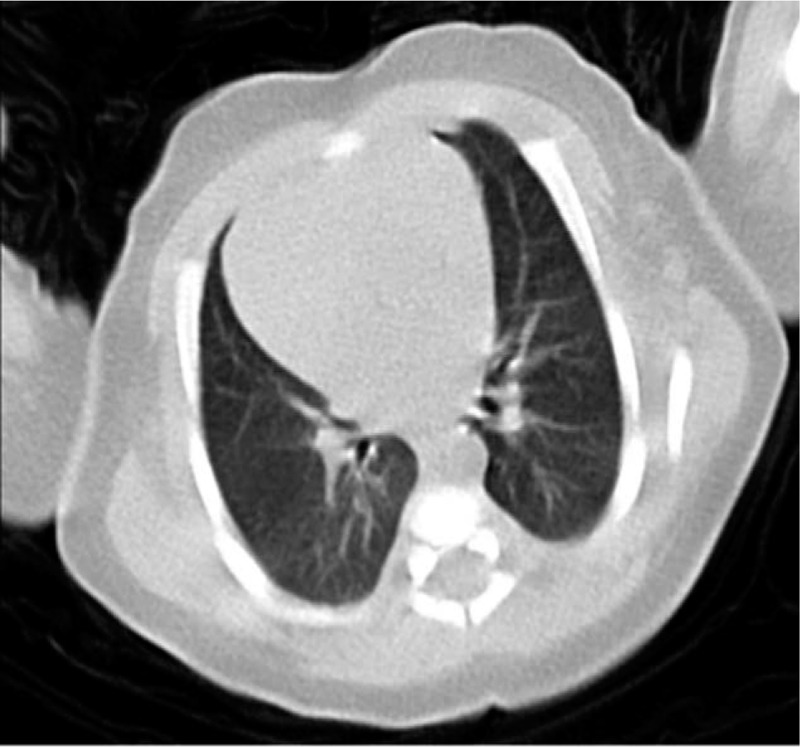
Chest computed tomographic images of the infant infected with SARS-CoV-2. No thoracic abnormalities were noted on February 12, 2020 (Illness Day 3). SARS-CoV-2 = severe acute respiratory syndrome coronavirus 2.

**Figure 3:**
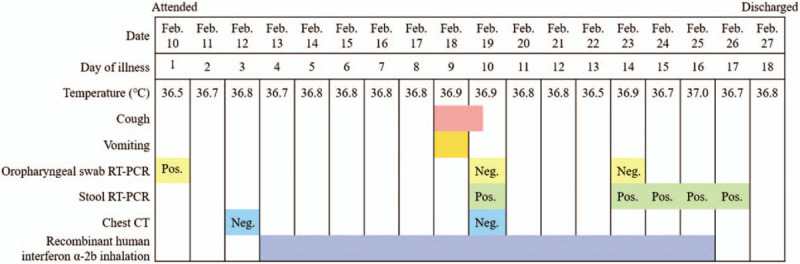
Maximum body temperatures, symptoms, RT-PCR assay for SARS-CoV-2, chest CT and treatment according to day of illness, February 10 to February 27, 2020. CT = computed tomography, RT-PCR = reverse transcription-polymerase chain reaction, SARS-CoV-2 = severe acute respiratory syndrome coronavirus 2.

A 35 day-old male infant had close contact with 2 confirmed patients of COVID-19, who were his grandmother and mother (Fig. 1). Then, the infant was admitted to a hospital on February 10, 2020, after his oropharyngeal swab tested positive for SARS-CoV-2 by reverse transcription-polymerase chain reaction (RT-PCR) (Sansure Biotech Inc. ( 680 Lusong Road, Changsha High-tech Industrial Development Zone, Hunan Province, China)) assay. Before admission, the infant had no fever, cough, dyspnea, listlessness, milk rejection, vomiting, diarrhea, and other symptoms. The infant's grandmother, who has lived in Ezhou City, Hubei Province, for a long time, flew from Wuhan in Hubei Province to Chengdu in Sichuan Province on January 20, 2020, and then she was picked up by her son from the airport to their home in Deyang, Sichuan Province. On February 1, 2020, his grandmother became febrile with temperature of 38.5°C. She was tested positive for COVID-19 with detectable SARS-CoV-2 from her oropharyngeal swab specimen by RT-PCR (Sansure Biotech Inc.), and bilateral ground-glass opacities were observed on chest computed tomography (CT). The mother of the infant had close contact with the grandmother and had developed fever, diarrhea, and myalgia after 4 days of contact on January 23, but the oropharyngeal swab specimens collected on that day showed no detectable SARS-CoV-2. The mother was kept in isolation at home for observation, and her symptoms improved remarkably within a short time after symptomatic treatment, including antidiarrheal drug and rehydration. On February 10, 2020, SARS-CoV-2 was detected from the oropharyngeal swab of the mother for routine reinspection, confirming the diagnosis of COVID-19, and was admitted to a hospital for further management. On admission, the sputum, oropharyngeal swab, and stool were tested positive, whereas the breast milk was tested negative for SARS-CoV-2 by RT-PCR. The infant's father, elder sister (16 years old), and elder brother (11 years old) had no fever and respiratory system or digestive system discomfort, and the tests for SARS-CoV-2, including CT scans and RT-PCR assay, were all negative. The infant's vital signs were stable on admission, and physical examination and chest CT showed no abnormality (Fig. 2). Laboratory investigations showed white blood cell count of 10.81 × 10^9^/L (normal, 8–12 × 10^9^/L), hemoglobin concentration of 115 g/L (normal, 110–160 g/L), high-sensitivity C-reactive protein of 0.34 mg/L (normal, 0.5–10 mg/L), creatinine concentration of 21.4 μmol/L (normal, 53–106 μmol/L), alanine aminotransferase of 18 IU/L (normal, 9–50 IU/L), and aspartate aminotransferase of 18 IU/L (normal, 15–40 IU/L). The feeding patterns of the infant were changed from breastfeeding to formula feeding because his mother was positive for COVID-19. His therapeutic schedule included aerosol inhalation of recombinant human interferon α-2b (1 million units twice a day) for 13 days and supportive therapy. On the seventh day of admission, the infant presented with cough and vomiting of milk. However, the symptoms of cough and vomiting were relieved in 1 day; thus, no adjustment was made on the treatment. The patient was tested negative for other pathogens including influenza A and B, parainfluenza (type 1 to 3), respiratory syncytial virus, adenovirus, and *Mycoplasma pneumoniae*. Reexamination of chest CT and oropharyngeal swab RT-PCR assay for SARS-CoV-2 showed no abnormality, but multiple reexaminations of stool showed positive results for SARS-CoV-2 RT-PCR assay until discharge. On the 17th day of admission, the infant had no fever or symptoms of respiratory or digestive system infection, and no abnormality was showed in chest CT. Two consecutive (1 day apart) oropharyngeal swabs tested negative for SARS-CoV-2, meeting the discharge criteria, and then the patient was discharged on February 27, 2020 (Fig. 3).

## 3 Discussion and conclusion

Recent data show that the prevalence of COVID-19 in children is relatively low. The proportion of children under the age of 18 and 10 accounts for 2.4% and 1% of all reported cases.^[4,5,9]^ In comparison with adults, most of the children diagnosed with COVID-19 experience mild symptoms, faster recovery, shorter virologic clearance time, and good prognosis.^[10]^ In this case, the infant was only 35 days old when he was tested positive for COVID-19, making him the youngest infant, except for newborn reported. He had no clinical symptoms at the beginning of his illness; thus, diagnosing was difficult without a definite history of epidemiological exposure. The number of infections in infants might be underestimated because of the mild symptoms or no symptoms.

Family clustering is an important epidemiological feature of the COVID-19 outbreak, which indicates that the virus is highly infectious. Based on existing epidemiological data, 56% (34/61) of children with COVID-19 demonstrated clear evidence of transmission through family gatherings.^[11]^ The infant is a second-generation case caused by intra-family transmission, suggesting that close family contact is the main way that infant will be infected by SARS-CoV-2, which is similar to the main transmission pattern of SARS and Middle East respiratory syndrome in children.^[7,8]^ Therefore, strengthening infant's virus screening in families with infected kins is important for early diagnosis, isolation, and treatment when symptoms are atypical.

Mild or asymptomatic COVID-19 may not result in adverse clinical outcomes, but the infectivity of infants with mild or asymptomatic COVID-19 should not be ignored because the infants may be a source of transmission in the community. In this case, the multiple reexaminations of stool of the infant and his mother showed positive for SARS-CoV-2 RT-PCR assay. Studies have proven that SARS-CoV-2 could be detected in the gastrointestinal tract, saliva, and urine,^[12,13]^ and these routes of potential transmission need to be further investigated. A study by Cai et al revealed that viral RNA from feces of children with COVID-19 was detected at a high rate, and the detoxification period can be as long as 2 to 4 weeks.^[14]^ Therefore, the management and disposal of patients’ stool need to be strengthened even after discharged. Previous studies have also shown that stool can be an excellent specimen for SARS diagnosis, particularly in the later stage of the illness, but data are lacking in children.^[15–17]^ Whether stool specimen test can be used as the diagnostic criteria of COVID-19 has to be further debated. In addition, infants younger than 1 year may not stand wearing masks; therefore, caregivers should strengthen their own protection, such as wearing masks and hand hygiene, to avoid infections because of close contact. Meanwhile, access to public places and group activities should be reduced or restricted; this measure in controlling the spread of COVID-19 has been proven effective in China. Wang et al. divided epidemic prevention and control in Wuhan into 3 stages and used the susceptible-exposed-infected-removed dynamic model to calculate the basic reproduction number (R0) and the effective reproduction number (*R*_*T*_). The first stage was the natural occurrence and spread of the epidemic from January 18 to January 23 with R0 = 3.31; the second stage was the blockade of Wuhan and the advocacy of residents going out less from January 23 to February 5 with *R*_*T*_ = 1.12; and the third stage was cabin hospitals put into use from February 6 to February 13 to ensure that all cases are admitted to hospitals and close contacts are under intensive medical observation with *R*_*T*_ = 0.71.^[18]^ The results showed the efficacy of the measures taken by China, which indicate the inflection point of the epidemic; thus, the incidence of the disease will gradually decrease until its disappearance. Other preventive measures include washing baby's hands frequently and cleaning and disinfecting their toys and tableware regularly. Further research is needed to fully understand the epidemiology and clinical characteristics of COVID-19 in infants to guide clinical decisions about diagnosis and treatment.

## Author contributions

**Project administration:** Gang Mai, Zhen Liu.

**Writing – original draft:** Wen Cao, Haoyuan Ren.

**Writing – review & editing:** Haoyuan Ren, Gang Mai.
